# Characterising the nature of the beast: Challenges associated with understanding patient safety within community-based mental health services

**DOI:** 10.1192/j.eurpsy.2022.1609

**Published:** 2022-09-01

**Authors:** P. Averill, C. Vincent, G. Reen, C. Henderson, N. Sevdalis

**Affiliations:** 1 King’s College London, Centre For Implementation Science, Institute Of Psychiatry, Psychology And Neuroscience, London, United Kingdom; 2 University of Oxford, Department Of Experimental Psychology, Oxford, United Kingdom

**Keywords:** Community Mental Health Services, Patient safety, Improvement science

## Abstract

**Introduction:**

Patient safety problems stemming from healthcare represent a significant cause of morbidity and mortality globally. The evidence base on safety in mental healthcare, particularly regarding community-based mental health services, has long fallen behind that of physical healthcare, with fewer research publications, developed primarily in isolation from the wider improvement science discipline. This disconnect both yields, and stems from, conceptual and practical challenges which must be surmounted in order to advance the science and improvement of safety in mental healthcare.

**Objectives:**

The objectives of this research were to conduct a narrative review to provide an overview of conceptual issues in this area, their origins, and implications for patient safety science and clinical care. We also sought to identify approaches to overcoming these issues.

**Methods:**

We examined theoretical and empirical evidence from the fields of patient safety, mental health, and improvement science to address this knowledge gap.

**Results:**

We identified challenges with defining safety in the context of community mental healthcare, ascertaining what constitutes a ‘preventable’ safety problem requiring intervention, and in finding relevant research evidence. The research indicated that risk management has taken precedence over proactive safety promotion in mental healthcare. This positions service users as the origin of safety risks, with iatrogenic harm and latent system hazards associated with mental healthcare widely overlooked.

**Conclusions:**

We propose a broader conceptualisation of safety to advance the field and outline potential next steps for the integration and uptake of different sources of ‘safety intelligence’ within community mental health services.

**Disclosure:**

NS is the director of London Safety and Training Solutions Ltd, which offers training in patient safety, implementation solutions and human factors to healthcare organisations and the pharmaceutical industry. The other authors have no competing interests.

